# CXCL8 secreted by immature granulocytes inhibits WT hematopoiesis in chronic myelomonocytic leukemia

**DOI:** 10.1172/JCI180738

**Published:** 2024-09-17

**Authors:** Paul Deschamps, Margaux Wacheux, Axel Gosseye, Margot Morabito, Arnaud Pagès, Anne-Marie Lyne, Alexia Alfaro, Philippe Rameau, Aygun Imanci, Rabie Chelbi, Valentine Marchand, Aline Renneville, Mrinal M. Patnaik, Valerie Lapierre, Bouchra Badaoui, Orianne Wagner-Ballon, Céline Berthon, Thorsten Braun, Christophe Willekens, Raphael Itzykson, Pierre Fenaux, Sylvain Thépot, Gabriel Etienne, Emilie Elvira-Matelot, Francoise Porteu, Nathalie Droin, Leïla Perié, Lucie Laplane, Eric Solary, Dorothée Selimoglu-Buet

**Affiliations:** 1 INSERM U1287, Gustave Roussy Cancer Center, Villejuif, France.; 2 Université Paris-Saclay, Faculté de Médecine, Le Kremlin-Bicêtre, France.; 3 INSERM U1018, Gustave Roussy Cancer Center, Villejuif, France.; 4 Institut Curie, PSL Research University, Paris, France.; 5 INSERM, U900, Paris, France.; 6 MINES ParisTech, CBIO–Centre for Computational Biology, PSL Research University, Paris, France.; 7 INSERM US23, CNRS UAR 3655, Gustave Roussy Cancer Center, Villejuif, France.; 8 INOVARION, Paris, France.; 9 Department of Medical Biology and Pathology, Gustave Roussy, Villejuif, France.; 10 Division of Hematology, Department of Internal Medicine, Mayo Clinic, Rochester, Minnesota, USA.; 11 Cellular Therapy Department, Gustave Roussy Cancer Center, Villejuif, France.; 12 Department of Hematology and Immunology, Henri-Mondor Hospital, AP-HP, Créteil, France.; 13 University of Paris Est Créteil, INSERM, IMRB, Créteil, France.; 14 Clinical Hematology Department, University Hospital, Lille, France.; 15 Clinical Hematology Department, Hôpital Avicenne, Bobigny, France.; 16 Hematology Department, Gustave Roussy Cancer Center, Villejuif, France.; 17 Adult Hematology Department, Saint-Louis Hospital, Assistance Publique-Hôpitaux de Paris, Paris, France.; 18 Université Paris Cité, Génomes, Biologie Cellulaire et Thérapeutique U944, INSERM, CNRS, Paris, France.; 19 Clinical Hematology Department, University Hospital, Angers, France.; 20 Clinical Hematology Department, Institut Bergonié, Bordeaux, France.; 21 Institut Curie, Université PSL, Sorbonne Université, CNRS UMR168, Laboratoire Physico-Chimie Curie, Paris, France.; 22 Institut d’Histoire et Philosophie des Sciences et des Techniques, CNRS U8590, Université Paris I Panthéon-Sorbonne, Paris, France.

**Keywords:** Hematology, Cytokines, Leukemias, Neutrophils

## Abstract

Chronic myelomonocytic leukemia (CMML) is a severe myeloid malignancy with limited therapeutic options. Single-cell analysis of clonal architecture demonstrates early clonal dominance with few residual WT hematopoietic stem cells. Circulating myeloid cells of the leukemic clone and the cytokines they produce generate a deleterious inflammatory climate. Our hypothesis is that therapeutic control of the inflammatory component in CMML could contribute to stepping down disease progression. The present study explored the contribution of immature granulocytes (iGRANs) to CMML progression. iGRANs were detected and quantified in the peripheral blood of patients by spectral and conventional flow cytometry. Their accumulation was a potent and independent poor prognostic factor. These cells belong to the leukemic clone and behaved as myeloid-derived suppressor cells. Bulk and single-cell RNA-Seq revealed a proinflammatory status of iGRAN that secreted multiple cytokines of which CXCL8 was at the highest level. This cytokine inhibited the proliferation of WT but not CMML hematopoietic stem and progenitor cells (HSPCs) in which CXCL8 receptors were downregulated. CXCL8 receptor inhibitors and CXCL8 blockade restored WT HSPC proliferation, suggesting that relieving CXCL8 selective pressure on WT HSPCs is a potential strategy to slow CMML progression and restore some healthy hematopoiesis.

## Abstract 2

In chronic myelomonocytic leukemia, blocking CXCL8 produced by clonal dysplastic granulocytes is a potential therapeutic strategy to slow disease progression

## Graphical abstract

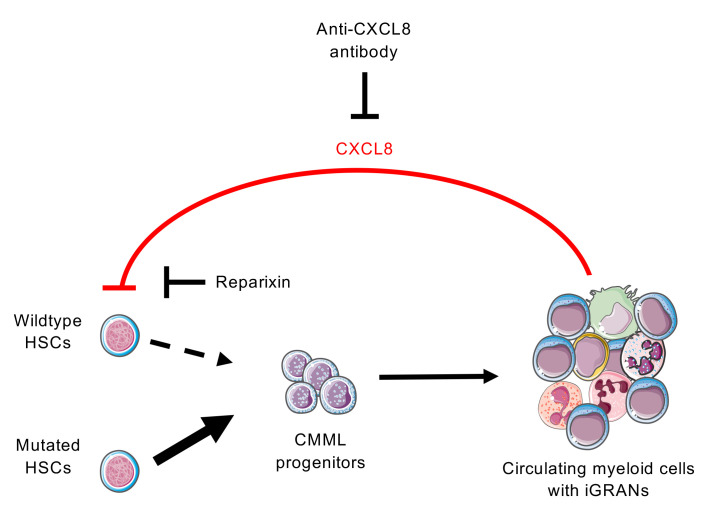

## Introduction

Chronic myelomonocytic leukemia (CMML) is a myeloid malignancy defined by overlapping features of both myeloproliferative and myelodysplastic neoplasms (1). CMML diagnosis is based on sustained absolute and relative peripheral blood (PB) monocytosis with abnormal partitioning of PB monocyte subsets, one or more clonal cytogenetic or molecular abnormalities, and/or dysplasia in at least one lineage (2). The disease predominantly affects elderly patients, and mutational signatures of leukemic cells suggest that aging is the main cause of the disease (3). Multiple studies have substantiated the clinical and molecular distinction of dysplastic (MD-CMML) and proliferative (MP-CMML) CMML on the basis of a cutoff WBC count of 13 × 10^9^/L (4). Blast cell count in the bone marrow also separates CMML in subgroups with distinct outcomes (5). A watch-and-wait attitude with careful monitoring is proposed for lower risk patients, while severe CMML requires therapy (1). Allogeneic stem cell transplantation, which is the only potentially curative treatment, is commonly precluded by age and comorbidities and only partially abrogates the risk of relapse (6). In patients with MD-CMML, hypomethylating agents can restore balanced hematopoiesis, but do not reduce the variant allele frequency (VAF) among circulating myeloid cells (3) and do not prevent progression to acute myeloid leukemia (AML) (1). In patients with MP-CMML, hypomethylating agents do not demonstrate a survival benefit compared with cytoreductive therapy with hydroxyurea (7). Therefore, there is an urgent need for additional therapeutic approaches for this disease.

CMML is a clonal disorder driven by the linear accumulation, in the hematopoietic stem cell (HSC) compartment, of somatic variants that diversely affect DNA methylation, histone modifications, pre-mRNA splicing, and cell signaling (4). Clonal architecture analyzed at the single-cell level indicates early clonal dominance with a very low number of residual WT HSCs in the bone marrow (8). Myeloid differentiation of mutated HSCs is amplified by hypersensitivity of myeloid progenitor cells to granulocyte/macrophage colony-stimulating factor (GM-CSF) (9). Virtually all the mature myeloid cells circulating in the body belong to the malignant clone (10, 11).

While 15%–30% of CMML patients die from disease transformation into acute leukemia, most of them demonstrate insidious physical exhaustion in an inflammatory climate. Circulating myeloid cells, predominantly monocytes and neutrophils, may contribute to the elevated levels of proinflammatory cytokines detected in the plasma of CMML patients (12, 13). Analysis of gene expression in PB monocytes indicates a proinflammatory phenotype (10, 14). In mouse models of chronic myeloid malignancies, inflammatory cytokines secreted by mature myeloid cells of the leukemic clone promote HSC expansion (15, 16). Therapeutic inhibition of these cytokines prevents disease development and progression (16, 17). Such feed-forward loops involving inflammatory cytokines produced by myeloid cells of the leukemic clone could contribute also to the progression of CMML, e.g., by promoting the expansion of mutated HSCs or slowing down that of WT cells.

Looking for the respective contribution of myeloid cell subsets of the leukemic clone to the inflammatory climate observed in CMML, we focused the present study on the granulocytic lineage. Neutrophil precursors, including metamyelocytes, myelocytes, and promyelocytes, which are normally retained in the bone marrow, are detected cytologically in the PB of a fraction of CMML patients and referred to as immature myeloid cells (IMCs) (13, 18). We used single-cell approaches to define immature granulocytes (iGRANs) and analyze further their contribution to disease outcome and pathogenesis. In 2 independent cohorts of patients, the presence of iGRANs in the PB correlates with a poor outcome. These cells, which share the same clonal origin as monocytes, demonstrate an inflammatory and immunosuppressive phenotype. They secrete high levels of CXCL8, a cytokine that inhibits the proliferation and differentiation of WT HSCs while sparing mutated HSCs in which CXCL8 receptors are downregulated. Reparixin, a clinical drug targeting the CXCL8-CXCR1/2 axis as well as a CXCL8 neutralizing antibody, restores the proliferation of WT HSCs. The feed-forward loop that involves CXCL8 produced by dysplastic granulocytes of the CMML leukemic clone in the repression of residual WT HSC suggests an innovative strategy for the therapeutic management of CMML patients.

## Results

### Identification and characterization of iGRANs.

We first assessed the prognostic significance of IMC detection of 1% or more on routine blood smears in a cohort of 580 consecutive CMML patients at diagnosis from Mayo Clinic (median age: 71 years [range 18–95]; 68% males, 32% females) (Supplemental Table 1; supplemental material available online with this article; https://doi.org/10.1172/JCI180738DS1). The overall survival (OS) of the 351 patients with IMC was significantly lower than that of the 229 patients without IMC (*P* < 0.0001), suggesting a poor prognostic factor. However, when WHO-defined criteria used for CMML stratification, namely WBC count and blast cell fraction, were considered together with IMC subgroups in multivariate analysis, IMC and blast cell count were no longer significant (Supplemental Figure 1A). Actually, IMC is not part of most scoring systems proposed in CMML, which may be related to the limited reproducibility of IMC quantification on routine complete blood counts (19). We explored whether flow cytometry phenotyping of PB cells could refine IMC identification and characterization.

**Figure 1:**
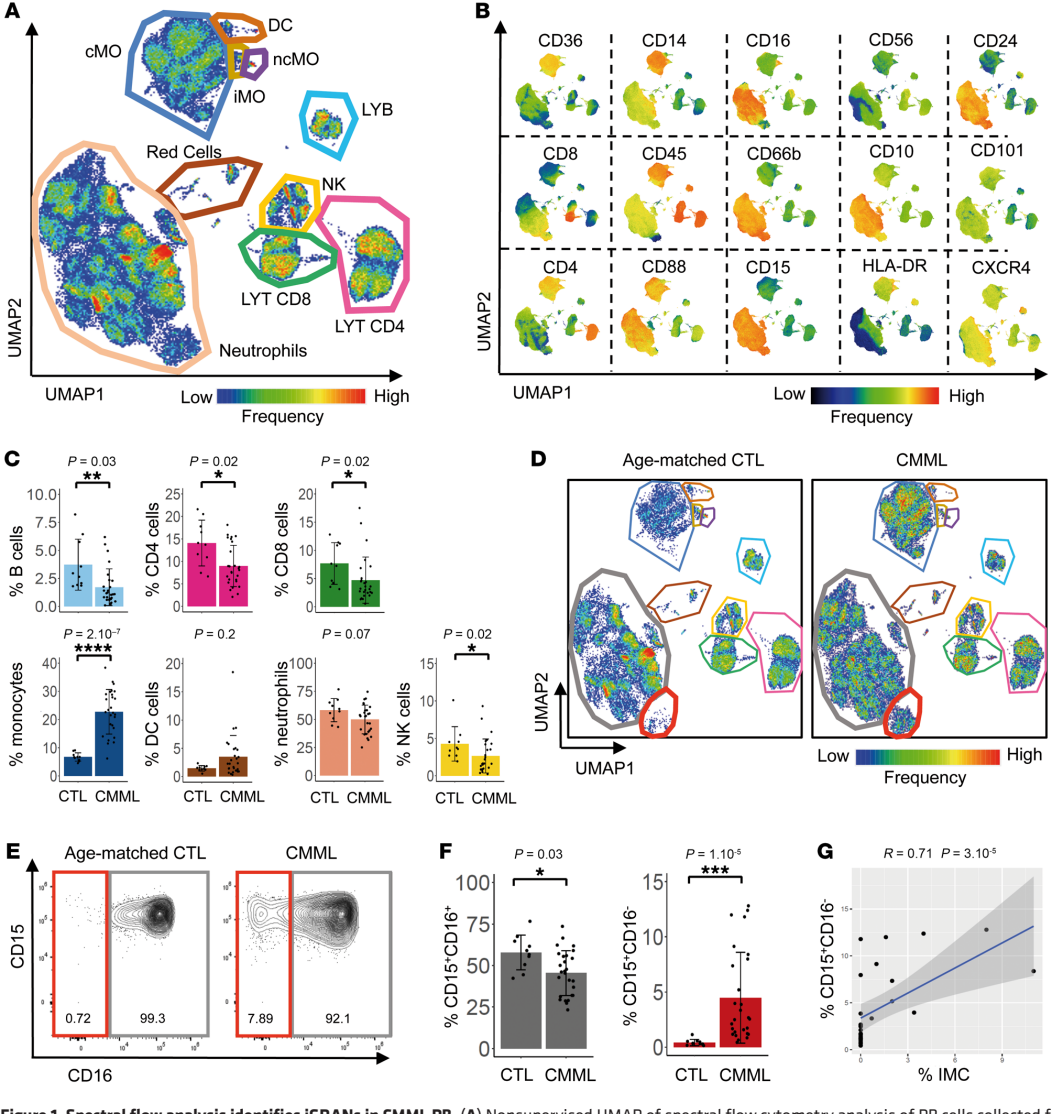
Spectral flow analysis identifies iGRANs in CMML PB. (**A**) Nonsupervised UMAP of spectral flow cytometry analysis of PB cells collected from 27 CMML patients and 10 age-matched control donors. cMO, classical monocytes; iMO, intermediate monocytes; ncMO, nonclassical monocytes; LYB, B lymphocytes; LYT, T lymphocytes. (**B**) Cell-surface expression of indicated markers on the UMAP shown in **A**. (**C**) Fraction of B, CD4^+^ T, CD8^+^ T, monocytes, DCs, neutrophils and NK cells among total CD45^+^ cells in age-matched controls (CTL) and CMML patients. Mann-Whitney *U* test. (**D**) Nonsupervised UMAP analysis of spectral flow cytometry data in the 10 controls compared with 27 CMML patients (60,000 cells for each condition). (**E**) Partition of neutrophil subsets based on CD15 and CD16 expression in each group. (**F**) Percentage of CD15^+^,CD16^–^ and CD15^+^,CD16^+^ neutrophils as separated in **E**, among CD45^+^ cells. Mann-Whitney *U* test. (**G**) Spearman’s correlation between CD15^+^CD16^–^ and IMC fractions in CMML PB. Adjusted *P* values are indicated above the graphs. *P < 0.05; **P < 0.01; ***P < 0.001; ****P < 0.0001.

We used spectral flow cytometry with a panel of 34 antibodies recognizing cell-surface markers (Supplemental Table 2) to generate an overview of cell populations in the PB. Pooled data from untreated CMML patients (*n* = 27) and age-matched controls (*n* = 10) (Supplemental Table 3) were subjected to a clustering analysis and visualized after a dimensionality reduction using the unsupervised uniform manifold approximation and projection (UMAP) algorithm (20) (Figure 1A and Supplemental Figure 1B). This approach allowed identifying classical, intermediate, and nonclassical monocytes; dendritic cells; B cells; CD4^+^ and CD8^+^ T cells; NK cells; and neutrophils (Figure 1B). A gating strategy was applied to precisely quantify each cell population in every sample among WBCs (CD45^+^ cells, Supplemental Figure 1, C–J). Examination of the cell-type profiles of samples from patients with CMML and controls revealed the increased fraction of circulating monocytes that defines CMML (Figure 1C), with a typical accumulation of classical monocytes at the expense of intermediate and nonclassical monocytes (Supplemental Figure 1K) (21). The fractions of circulating B cells, CD4^+^ and CD8^+^ T cells, and NK cells decreased in CMML patients compared with age-matched controls, whereas no significant changes were observed in the global dendritic cell and granulocyte populations (Figure 1C).

A detailed analysis of neutrophils detected a cluster of cells in CMML samples that was hardly observed in age-matched controls (Figure 1D). Compared with neutrophils, this cell subset expressed higher levels of CD15, CD24, and CXCR4; lower levels of CD45, CD10, and CD101; and almost no CD16, suggestive of immature granulocytes (iGRANs, Figure 1B). Quantification of the neutrophil populations among CD45^+^ cells in individual samples revealed a significant decrease in the fraction of mature CD15^+^CD16^+^ neutrophils in the CMML group compared with the healthy donor group, while the fraction of CD15^+^CD16^–^ cells significantly increased with high interpatient heterogeneity (Figure 1, E and F). In the CMML group, the fraction of CD15^+^CD16^–^ neutrophils correlated with the IMC fraction detected on routine blood smears (*r* = 0.71, Figure 1G). Altogether, spectral flow cytometry analysis of CMML PB cells refined the detection of IMC and characterized a subset of cells as CD15^+^CD16^–^CD66b^+^ iGRANs.

### Flow cytometric detection of iGRANs refines CMML prognostication.

Having identified informative phenotypic markers by spectral flow cytometry analysis, we developed a conventional, multiparametric flow cytometry assay (Supplemental Table 2) to routinely quantify the fraction of iGRANs in freshly collected blood samples and revisit the prognostic significance of this parameter in CMML. We noticed that iGRANs were part of the PBMC population sorted by low-density gradient centrifugation, a process that removes a majority of CD15^+^CD16^+^CD66b^+^ mature neutrophils without affecting the iGRAN population (Supplemental Figure 2, A and B). Based on this low-density cell property, the conventional flow cytometry assay was performed on PBMCs (Supplemental Figure 2, C–J). The iGRAN fraction measured among Lin^–^CD16^–^CD11b^+^CD33^+^ cells correlated with that among total CD45^+^ cells (Supplemental Figure 2K). With this conventional flow cytometry assay, repeated measures of the iGRAN fraction over up to 1 year in 17 untreated CMML patients showed reproducible results (Supplemental Figure 2L).

**Figure 2:**
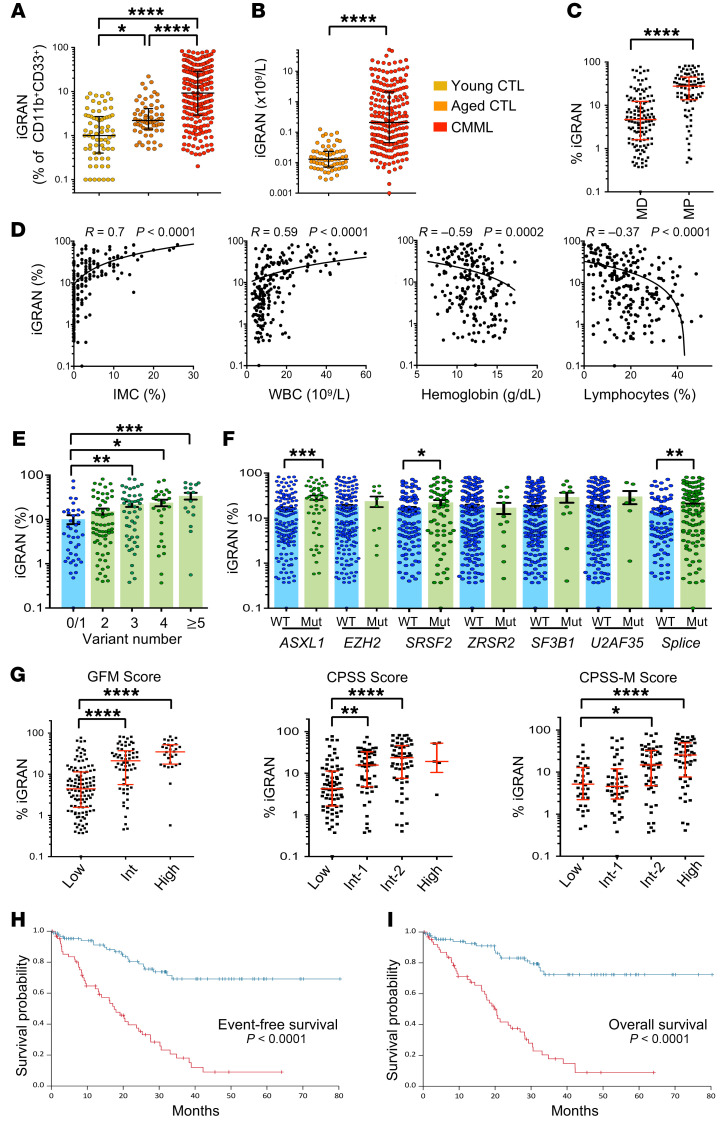
Elevated iGRAN fraction in the PB of CMML patients is a poor prognostic factor. (**A**) iGRAN fraction in CD11b^+^CD33^+^ population as measured by conventional flow cytometry in the PB of young controls (*n* = 71), age-matched controls (*n* = 64), and CMML patients (*n* = 209). Kruskal-Wallis test. (**B**) iGRAN absolute number (×10^9^/L) was measured in the PB of CMML patients compared with age-matched controls. Mann-Whitney *U* test. (**C**) iGRAN fraction in MD-CMML and MP-CMML subtypes according to the WHO classification. Mann-Whitney *U* test. (**D**) Spearman’s correlation between iGRAN fraction and IMC fraction, WBC count, hemoglobin level, and lymphocyte fraction in the PB of CMML patients. (**E**) iGRAN fraction in CMML patients grouped according to the number of mutations detected in a panel of 25 genes. Kruskal-Wallis nonparametric test. (**F**) iGRAN fraction in CMML patients grouped according to the mutational status of each indicated gene: WT or mutated (Mut). Splice: SRSF2+ZRSR2+U2AF1+SF3B1. Mann-Whitney *U* test. (**G**) iGRAN fraction in CMML patients grouped according to GFM, CPSS, and CPSS-M prognostic scores. Kruskal-Wallis test. (**H** and **I**) EFS (defined as time between diagnosis and AML transformation, death, or last follow-up) (**H**) and OS (time between diagnosis and death) (**I**) of CMML patients with high (≥14%, *n* = 66, in red) or low (<14%, *n* = 88, in blue) iGRAN fraction; log-rank test. *P < 0.05; **P < 0.01; ***P < 0.001; ****P < 0.0001.

Once calibrated, this flow cytometry assay was prospectively assessed between March 2015 and April 2019 on a learning cohort of 209 untreated CMML patients (median age: 75 years [range, 50–93] 63% males, 37% females) compared with 64 age-matched controls (median age: 74 years [range, 65–94]) and 71 younger healthy donors (<65 years old) (Supplemental Table 3). CMML diagnosis was supported by flow cytometry analysis of peripheral monocyte subsets (21): in 192 patients (92%), classical monocytes represented more than 94% of total monocytes, while a decreased fraction of slan^+^ nonclassical monocytes was observed in 15 of the 17 remaining patients (7%) (22, 23). The fraction of iGRANs among myeloid cells was significantly higher in aged controls (median 2.2% [range, 0.5%–22%]) than in younger ones (median 1.0% [range, 0%–0.9%], *P* = 0.0001) and was further increased in CMML patients (median 9.2% [range, 0.03%–82.5%], *P* < 0.0001) (Figure 2A). The absolute number of circulating iGRANs also was statistically increased in CMML patients, sometimes reaching values close to 50 × 10^9^/L, representing a 1,000-fold increase over the values in aged controls (Figure 2B). The fraction of iGRANs was significantly higher in MP-CMML than in MD-CMML (Figure 2C) and positively correlated with the fraction of IMCs quantified on blood smears (*r* = 0.7, *P* < 0.0001). However, the flow cytometric quantification of iGRANs has a more precise detection threshold for patients with low IMC (Figure 2D). The fraction of iGRANs also correlated with WBC count and with lower hemoglobin levels and lymphocyte fractions (Figure 2D). In contrast, no correlation was observed for iGRAN fraction with age, sex, presence of cytogenetic abnormalities, or total neutrophil fraction, and the correlations with monocyte fraction, platelet count, and WHO-defined subtypes remained weak (Supplemental Figure 3, A–E). Analysis of mutations in a panel of 35 genes recurrently mutated in CMML (Supplemental Table 3) showed an increase in the iGRAN fraction with an increasing number of mutated genes as well as with the presence of *ASXL1* or *SRSF2* gene mutations (Figure 2, E and F). No significant association was detected with the other common variants in CMML, including those in signaling genes (Supplemental Figure 3F). Finally, the iGRAN fraction increased with disease risk level, as measured using the Groupe Francophone des Myélodysplasies score (GFM) (24), the CMML-specific prognostic scoring system (CPSS) (25), and the CPSS-molecular (CPSS-M) score, which incorporates molecular genetic data (26) (Figure 2G). Together, these results indicate that iGRAN accumulation in blood is associated with poor prognosis scores and with poor prognosis markers like *ASXL1* mutations (27) or proliferative forms of the disease. The relationship between the iGRAN fraction and other parameters was validated by analyses performed using the absolute number of circulating cells (10^9^/L) (Supplemental Table 4), indicating the robustness of iGRAN quantification to separate the most severe forms of the disease.

Among the 209 patients included in the learning cohort, iGRAN quantification had been performed for 154 patients at diagnosis, i.e., ± 6 months from initial bone marrow examination. With a median follow-up of 34 months, 27 patients among 154 progressed to AML, and 62 died. The median OS and median event-free survival (EFS, defined as the time between diagnosis and AML transformation, death, or last follow-up) were 20.6 and 20 months, respectively. Univariate and multivariate Cox models were built with continuous iGRAN percentages and absolute numbers. The multivariate analysis model included clinical and mutation variables with independent prognostic value, based on the GFM scoring system, which includes age, WBC, hemoglobin level, platelet count, and *ASXL1* mutation (24). Both univariate and multivariate models showed a significant impact of iGRANs measured at diagnosis on EFS or OS (Supplemental Table 5). We then computed the data to dichotomize continuous variables by selecting cutoff values to maximize the log-rank statistics and picked out an iGRAN fraction of 14% or more of circulating myeloid cells or an absolute iGRAN number of 0.4 × 10^9^/L or more as optimal values to identify the impact of iGRANs on EFS and OS (Figure 2, H and I, and Supplemental Figure 3G). With these cutoff values, the iGRAN fraction improved the accuracy of existing prognostic scores, after adjusting for potential confounders (hazard ratio for iGRAN fraction in multivariate model: 3.678, 95% CI: [1.850–7.305] for OS and 3.003, 95% CI: [1.592–5.663] for EFS) (Supplemental Table 5). An increased number and fraction of iGRAN was also detected in a validation cohort of 160 CMML patients (Supplemental Table 3 and Supplemental Figure 3H), especially those with a proliferative phenotype (Supplemental Figure 3I), correlating again with IMC fraction, WBC, hemoglobin level, and lymphocyte fraction (Supplemental Figure 3J). Follow-up (median, 36.6 months) was available for 110 of these patients, of whom 13 progressed to AML and 43 died. Using cutoff values defined within the learning cohort (iGRAN fraction ≥14%, iGRAN number ≥0.4 × 10^9^/L), the impact of iGRANs on EFS and OS was validated (Supplemental Figure 3K).

Together, flow cytometric measurement of an iGRAN fraction of 14% or more of circulating myeloid cells or an absolute number of iGRAN of 0.4 × 10^9^/L or more is an independent biomarker of CMML severity.

### iGRANs are myeloid-derived suppressive cells.

**Figure 3:**
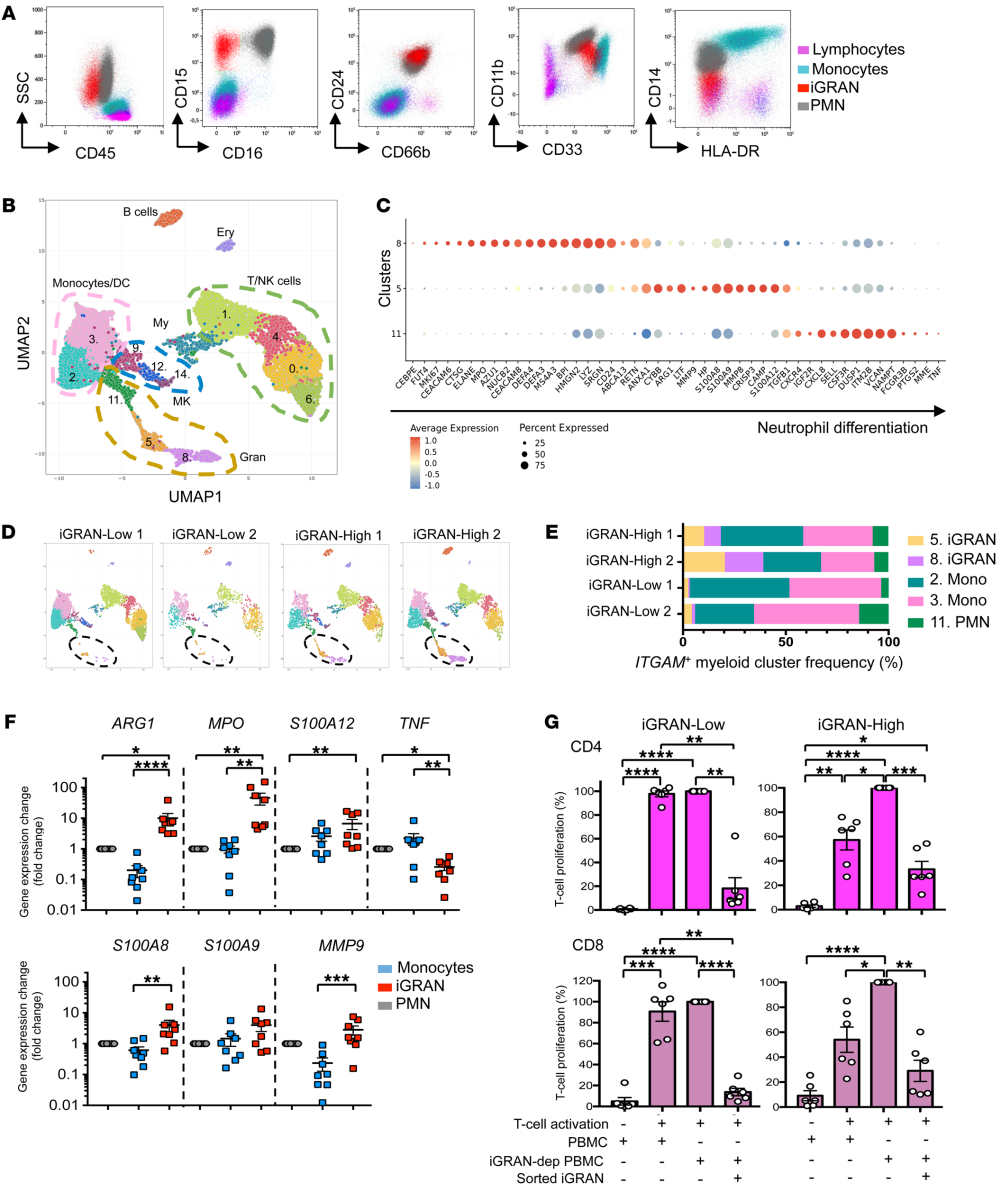
iGRANs demonstrate features of myeloid-derived suppressive cells. (**A**) Representative conventional flow plots showing cell-surface expression of indicated markers among low-density peripheral blood cells (PBMC), separating iGRANs from monocytes, residual PMN, and lymphocytes, according to the gating strategy shown in Supplemental Figure 2. (**B**) Single-cell analysis of PBMCs collected from 2 CMML patients with a fraction of 14% or more and 2 with a lower iGRAN fraction; unsupervised clustering of pooled data separating 15 cluster groups in indicated cell categories. Ery, erythroid cells; My, myeloid cells; MK, megakaryocytes; Gran, granulocytes. (**C**) Dot plot showing the average expression (color scaled) of selected granulocyte genes and the percentage of cells that expressed those genes in indicated granulocytic clusters. (**D**) UMAP representation of each patient sample, 2 iGRAN-low (<14%) and 2 iGRAN-high (≥14%) CMML patients. (**E**) Fraction of each cell type in ITGAM^+^ clusters corresponding to CD11b^+^CD33^+^ cells per CMML patient. Colors are cluster codes defined in **B**. (**F**) Expression of indicated genes in enriched fraction of iGRANs, monocytes, and neutrophils measured by RT-qPCR and normalized to *RPL32*, *GUS*, and *GAPDH* housekeeping genes (*n* = 8 CMML patients). Kruskal-Wallis test. (**G**) Suppressive activity of iGRANs on T cell proliferation. iGRAN-low (<14%, *n* = 6, left panels) and iGRAN-high (≥14%, *n* = 6, right panels) PBMCs were labeled with Cell Trace Violet before activating T cells with anti-CD3 and anti-CD28 antibodies; CD4 (upper panels) and CD8 (lower panels) T cell proliferation was measured at day 4 by flow cytometry. We used PBMCs without any manipulation, PBMCs in which iGRAN have been depleted (iGRAN-dep PBMC), and iGRAN-dep PBMCs with addition of 10% sorted iGRANs. T cell proliferation (%) is relative to the highest proliferation, observed with iGRAN-dep PBMCs. One-way ANOVA, Tukey’s multiple comparison. *P < 0.05; **P < 0.01; ***P < 0.001; ****P < 0.0001.

Having identified the poor prognostic significance of iGRAN excess, we next sought to assess their functional impact. The phenotype of these cells (CD45^lo^, CD33^+^, CD11b^+^, HLA-DR^–^, CD14^–^, CD15^+^, CD24^+^, CD66b^+^, and low density) suggested that they may be granulocytic myeloid-derived suppressor cells (G-MDSCs, also referred to as PMN-MDSCs) (Figure 3A) (28). To explore this possibility, we selected 2 patients with an iGRAN-high (≥14%) and 2 patients with an iGRAN-low fraction and performed single-cell RNA-Seq analysis (scRNA-Seq) of PBMCs. The data were pooled and subjected to dimensionality reduction using the UMAP algorithm, and 15 clusters were identified (Figure 3B). Based on the expression of marker genes, the cell types of clusters were identified, including T/NK cells (*CD3E*, *NKG7*), B cells (*CD79*A), erythroid cells (*HBA1*), megakaryocytes/platelets (*PF4*), monocytes (*CD14*, *CD33*), and granulocytes (*FCGR3B*, *S100A9*, *S100A8*, and *LYZ*) (Supplemental Figure 4, A–C). Granulocytes encompassed several clusters (clusters 11, 5, and 8), corresponding to sequential stages of neutrophil maturation. Cells in cluster 8 expressed genes encoding primary neutrophil granule proteins, such as *ELANE*, *CEACAM*, *AZU1*, *DEFA1*, and *DEFA3*, indicating promyelocytes and myelocytes (Figure 3C and Supplemental Figure 4B). Cells in cluster 5 expressed genes encoding secondary and tertiary neutrophil granule proteins, such as *MMP9*, *MMP8*, and *LTF*, indicating metamyelocytes. Lastly, cells in cluster 11 expressed high levels of genes that characterize mature neutrophils, such as *MME* (CD10) and *FCGR3B* (CD16). Patients with an iGRAN-high fraction showed strong enrichment of cells in clusters 5 and 8 (Figure 3, D and E), validating the initial description of this population as iGRANs.

The gene expression profile of human G-MDSCs distinguishes them from mature neutrophils or monocytes (28). This profile was recapitulated in clusters 5 and 8, which exhibited high expression of cell cycle genes such as *MKI67* as well as high expression of *ARG1*, *MPO*, *S100A8*, *ANXA1*, *CYBB*, and *S100A12* genes and did not express the *TNF* gene (Figure 3B). This gene signature was validated by reverse transcription quantitative PCR (RT-qPCR) analysis in sorted CD16^+^ neutrophils, CD14^+^ monocytes, and CD15^+^CD16^–^ iGRANs collected from 8 CMML patients. iGRANs expressed significantly higher levels of *ARG1*, *S100A12*, and *MPO*; similar levels of *S100A8*, *S100A9*, and *MMP9*; and lower levels of *TNF* compared with mature neutrophils or monocytes (Figure 3F). These features are consistent with the hypothesis of G-MDSC (28, 29).

The main characteristic of G-MDSCs is their ability to suppress immune cells. Consistent with the spectral flow cytometry analyses showing a significant inverse correlation between the iGRAN and CD4^+^ T cell fractions (*r* = –0.49, *P* = 0.01, Supplemental Figure 4D), conventional flow cytometry analysis showed an inverse correlation between the iGRAN and lymphoid cell fractions (obtained by WBC count). We also cultured PBMCs collected from untreated iGRAN-low (<14%) and iGRAN-high (≥14%) CMML patients for 4 days with anti-CD3 and anti-CD28 antibodies for T cell activation. iGRAN depletion from iGRAN-high, but not iGRAN-low PBMCs increased CD4^+^ or CD8^+^ T cell proliferation. When a fixed ratio of sorted iGRANs was added to iGRAN-depleted PBMCs, T cell proliferation was strongly reduced in all situations, whatever the initial fraction of iGRANs in PBMCs (Figure 3G and Supplemental Figure 4, E and F). Taken together, these results demonstrate that iGRANs are G-MDSCs.

### iGRANs are clonal cells with high inflammatory activity.

**Figure 4:**
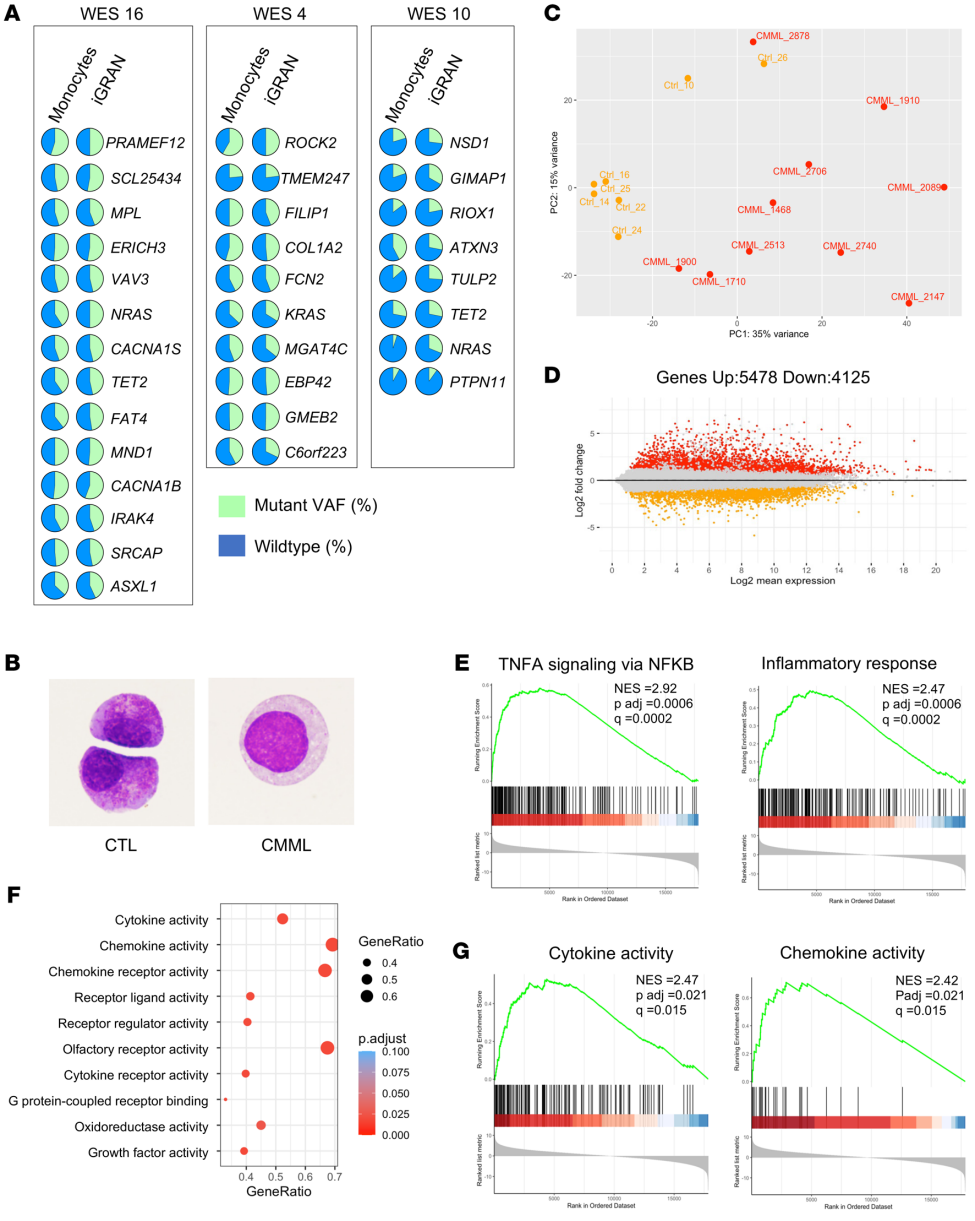
iGRANs are clonal, proinflammatory granulocytes. (**A**) VAF of indicated somatic variants detected by whole exome sequencing of sorted monocytes (left) and iGRANs (right) in 3 CMML patients (WES numbers: 16, 4, 10). (**B**) May-Grünwald-Giemsa staining of iGRAN sorted from a control donor (CTL) and a CMML patient sample. Original magnification, ×1,000. (**C**–**G**) Bulk RNA-Seq of sorted iGRANs collected from 7 healthy donors (CTRL) and 10 CMML patients; PCA of regularized logarithm (rlog) transformed data based on the top 500 varying genes using plotPCA function of the DESeq2 package (**C**). MA plot of differentially expressed genes between iGRANs collected from control and CMML patients (**D**). GSEA of indicated hallmark pathways enriched in CMML versus control (**E**). Top10 nominal enrichment score of pathways involving upregulated genes in CMML versus control cells according to GO molecular function (**F**). GSEA of indicated hallmark pathways enriched in CMML versus control (**G**). Adjusted *P* and *q* values are indicated on the graphs.

G-MDSCs were shown to be part of the leukemic clone in AML (30) and in chronic myeloid leukemia (31). Ambiguity remains in myelodysplastic neoplasms (32), and the question has never been addressed in CMML. The early clonal dominance depicted in this disease (8) left little room to the hypothesis of nonmutated granulocytic differentiation, but a subclonal expansion was possible. To explore the link between iGRANs and the somatic genetic abnormalities that characterize CMML, we performed whole-exome sequencing of sorted iGRANs, monocytes, and T cells from 14 untreated CMML patients. For each patient analyzed, the somatic variants identified in iGRANs matched those detected in monocytes with similar VAFs (Figure 4A and Supplemental Table 6). These results indicate that iGRANs belong to the leukemic clone and their accumulation is not related to subclonal coding region variants.

To further characterize CMML-associated clonal iGRANs, we compared them with sorted CD15^+^CD16^–^ cells collected from healthy donors by cytapheresis after mobilization. Cytological examination validated the immature morphology of collected cells, corresponding to promyelocytes or myelocytes and revealed greater dysplasia in CMML iGRANs, which showed a loss of cytoplasmic granules and less condensed nuclear chromatin (Figure 4B). Transcriptomic profiles were studied in CD15^+^CD16^–^ cells sorted from 7 healthy donors and 10 untreated CMML patients. Gene expression in the 17 pooled samples was ranked. Genes with highest expression included *DEFA1*, *DEFA3*, *S100A9*, *MPO*, and *LYZ* (Supplemental Figure 5A). These genes were similarly expressed in iGRANs sorted from CMML and control samples, indicating that the cells under comparison belong to the same differentiation stage in the granulocytic lineage (Supplemental Figure 5B). Together, these analyses validated the granulocytic and immature phenotype of the sorted cells.

Principal component analysis (PCA) showed that patient and control cells mostly clustered separately (Figure 4C and Supplemental Figure 5C). Based on an adjusted *P* value of less than 0.05 and a log_2_-fold change greater than 2, a total of 9,603 genes were found to be differentially expressed in CMML and control samples, of which 5,478 were upregulated (Figure 4D). Pathway analysis using gene set enrichment analysis (GSEA) for hallmarks identified 33 pathways enriched in CMML iGRANs. The pathways with the highest enrichment scores were TNFA signaling via NF-κB, inflammatory response, and interferon response, whereas various metabolic pathways were downregulated (Figure 4E and Supplemental Figure 5D). GSEA for molecular function analysis showed the significant enrichment of gene sets associated with receptor binding and cytokine and chemokine activity in CMML-associated iGRANs (Figure 4, F and G). These results indicate a proinflammatory status of clonal iGRANs in CMML patients.

### CXCL8 is the main cytokine secreted by iGRANs.

**Figure 5:**
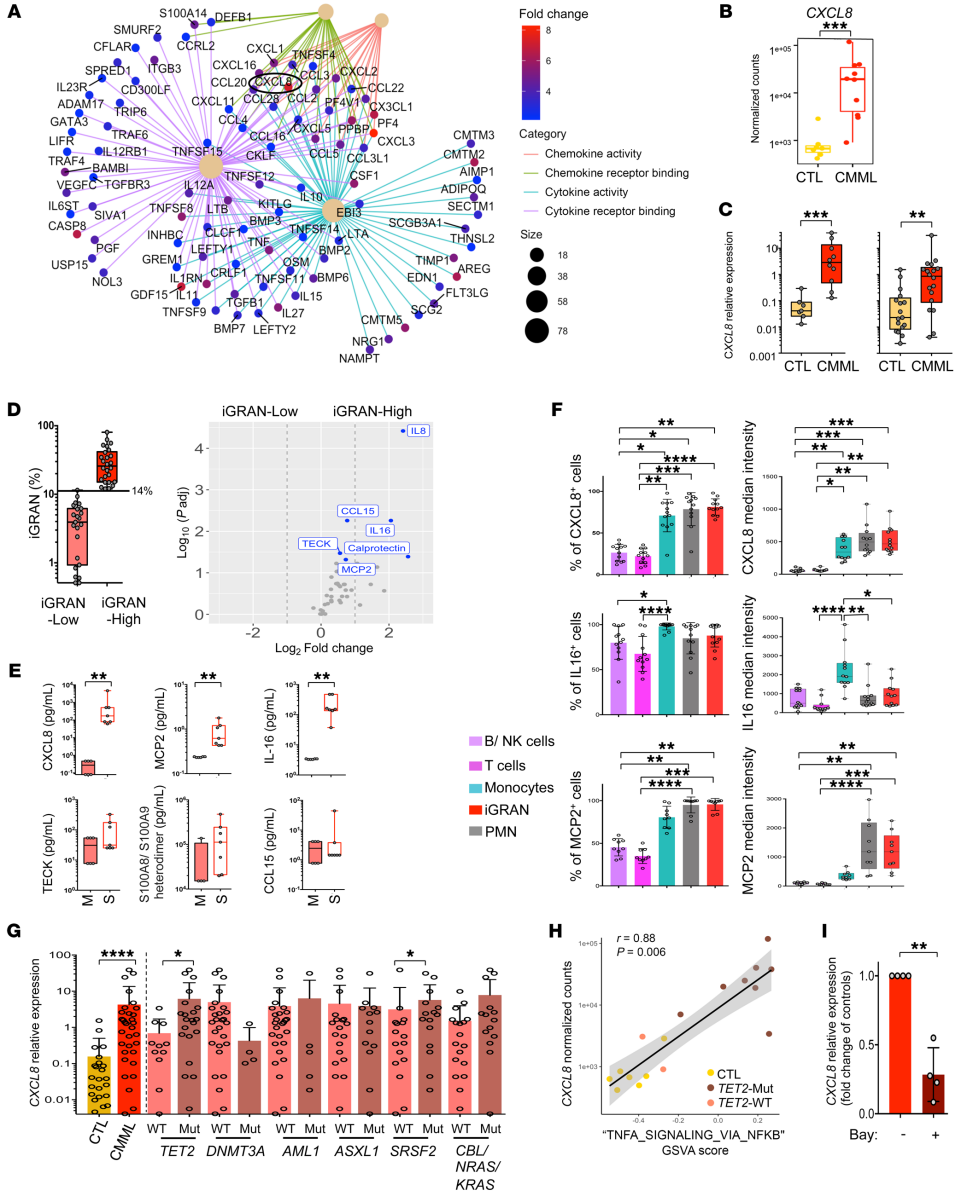
CXCL8 is one of the main cytokines released by CMML patient iGRANs. (**A** and **B**) Bulk RNA-Seq of sorted iGRAN collected from healthy donors (CTL) and CMML patients. Gene-concept network representation of enriched genes of 4 pathways (chemokine activity, chemokine receptor binding, cytokine activity, cytokine receptor binding). Dot color, log_2_foldchange. Size of beige central dots, number of core enriched genes (**A**). *CXCL8* mRNA expression (normalized counts) in control cells (*n* = 7) and CMML iGRAN (*n* =10) (**B**). Mann-Whitney *U* test. (**C**) *CXCL8* mRNA expression assessed by RT-qPCR in healthy donor and CMML; left panels, samples used for RNA-Seq; right panel, independent cohort of iGRANs collected from 17 control and 18 CMML. Ct values normalized to *GAPDH*, *RPL32*, and *GUS* genes. Mann-Whitney *U* test. (**D**) Volcano plot of cytokine and chemokine levels (*n* = 44) measured in circulating plasma of 23 iGRAN-low compared with 26 iGRAN-high CMML (threshold ≥14%, iGRAN fraction in box plot for each group on the left panel). (**E**) Indicated proteins were quantified in the supernatant of iGRANs (S), using culture medium (M) as a control. Mann-Whitney *U* test. (**F**) Intracellular cytokine production by B/NK cells, T cells, monocytes, iGRAN, and neutrophils (PMN) in fresh PB samples collected from CMML patients. Left panels, fraction of cells expressing the studied cytokine; right panels, median fluorescence intensity for CXCL8 (*n* = 12), IL-16 (*n* = 12), or MCP2 (*n* = 9) in cells expressing the cytokine. One-way ANOVA, Tukey’s multiple comparison. (**G**) *CXCL8* mRNA expression (RT-qPCR) in iGRAN of 17 healthy donors and 32 CMML, according to WT and mutated (Mut) status of indicated genes. Mann-Whitney *U* test. (**H**) Spearman’s correlation between *CXCL8* normalized expression and gene set variation analysis (GSVA) score for the hallmark “TNFA_SIGNALING_VIA_NFKB”. Each dot represents iGRAN sample from *TET2*-mutated patients (*n* = 8), *TET2*-WT patients (*n* = 2), and controls (*n* = 7). (**I**) *CXCL8* mRNA expression after 24 hours of iGRAN culture with 0.5 μM Bay 11-7082, normalized to *RPL32*, *HPRT*, and *PPIA* genes. Fold change compared with DMSO-treated iGRANs. Paired *t* test. *P < 0.05; **P < 0.01; ***P < 0.001; ****P < 0.0001.

Analysis of individual cytokine-encoding genes identified the *CXCL8* gene (C-X-C motif chemokine ligand 8, also known as interleukin-8 or IL-8) as the most highly overexpressed (Figure 5, A and B). *CXCL8* overexpression was confirmed by RT-qPCR analysis of 2 different sets of control and CMML cells (Figure 5C). Then we measured the plasma levels of 44 cytokines and chemokines in iGRAN-low (*n* = 23) and iGRAN-high (*n* = 26) CMML patients and found that CXCL8 was the cytokine present at the highest level in the plasma of iGRAN-high CMML patients (Figure 5D). C-C motif chemokine ligand 15 (CCL15), IL-16, the S100A8/S100A9 heterodimer (also known as calprotectin), MCP2 (monocyte chemoattractant protein 2, also known as CCL8), and TECK (CCL25) were also detected at significantly higher levels in the plasma of the iGRAN-high patient group compared with the iGRAN-low patient group (Figure 5D and Supplemental Figure 5E). Importantly, CXCL8, CCL15, the S100A8/S100A9 complex, and IL-16 plasma levels correlated with the iGRAN fraction detected by flow cytometry in CMML PB (Supplemental Figure 5F). Of these cytokines, CXCL8, IL-16, and MCP2 were those detected at high concentrations in the culture supernatant of sorted CMML-associated iGRANs (Figure 5E). By performing intracellular staining of fresh PB samples including neutrophils, we detected these 3 cytokines in both mature (neutrophils) and immature (iGRAN) granulocytes. IL-16 was more abundant in monocytes that also produce CXCL8, but low levels of MCP2 (Figure 5F). A significant correlation was observed between the fraction of iGRAN in CD11b^+^CD33^+^ cells and CXCL8 median fluorescence intensity in mature neutrophils (Supplemental Figure 5G). *CXCL8* expression, as measured by RT-qPCR in sorted iGRANs, was increased in CMML compared with healthy donor iGRANs. When correlated with somatic variant status, this increase was significant in *TET2*- and *SRSF2*-mutated samples (Figure 5G). As *CXCL8* gene expression was not increased in the only 4 *SRSF2*-mutated samples that were also WT for *TET2*, we focused on the role of *TET2* mutation in increasing *CXCL8* expression in iGRANs (Figure 5G). We observed an increased intracellular staining of CXCL8 in *TET2*-mutated compared with WT iGRANs (Supplemental Figure 5H). In accordance with the role of NF-κB in the transcriptional regulation of *CXCL8* gene (33, 34), the hallmark_TNFA_SIGNALING_VIA_NFKB pathway was enriched in *TET2*-mutated iGRANs (Figure 5H), as described in *TET2*-mutated neutrophils (35). Finally, *CXCL8* expression was decreased in iGRANs treated with the NF-κB pharmacological inhibitor Bay11-7082 for 24 hours (Figure 5I). Together, these data argue for a link between *CXCL8* overexpression in iGRANs and *TET2* mutation through NF-κB pathway activation.

### CXCL8 inhibition restores the growth of WT CD34^+^ cells.

**Figure 6:**
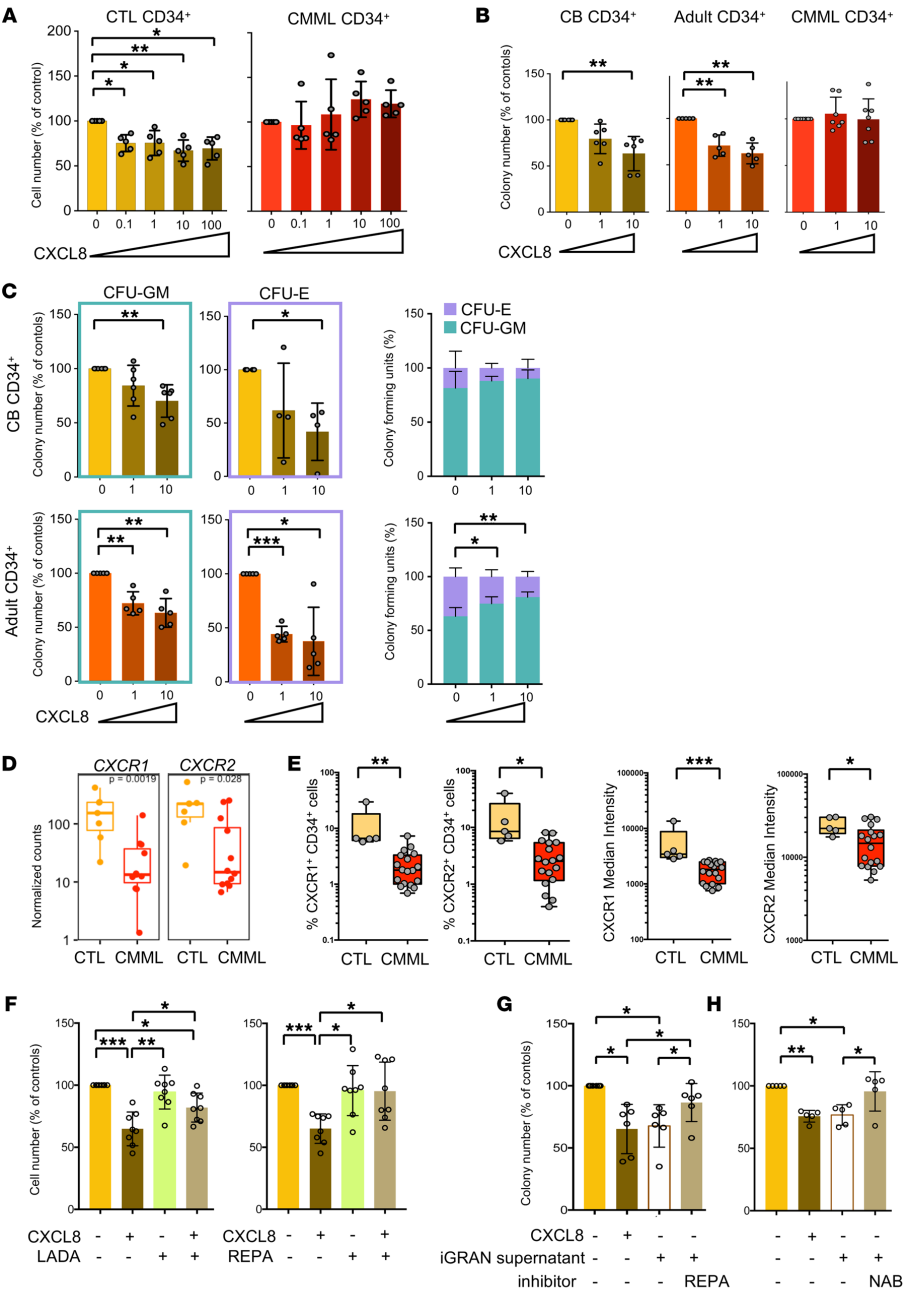
CXCL8 specifically inhibits the growth of WT CD34^+^ cells. (**A**) Cell output of CMML CD34^+^ cells in liquid culture for 3 days in the presence of indicated doses of CXCL8 (ng/ml); ratio to untreated samples. Data are represented as means ± SD. *n* = 5 per group. One-way ANOVA, Dunnett’s multiple comparison. (**B**) Total colony output of CD34^+^ cells cultured in methylcellulose in the presence of indicated doses of CXCL8 (ng/ml) for 14 days. CB, cord blood (*n* = 6). Adult, healthy donor bone marrow CD34^+^ cells (*n* = 5) or CMML samples (*n* = 7); ratio to untreated samples. Data are represented as means ± SD. One-way ANOVA, Dunnett’s multiple comparison. (**C**) Cord blood (*n* = 6 upper panels) and healthy donor bone marrow (*n* = 5 lower panels) CD34^+^ cells were cultured in methylcellulose for 14 days to generate CFU-GM (left panel) and CFU-E (middle panel) colonies in the absence or presence of indicated concentrations of CXCL8. Output of treated relative to untreated cells. Right panel, fractions of CFU-GM and CFU-E were represented together. Data are represented as means ± SD. One-way ANOVA, Dunnett’s multiple comparison. (**D**) *CXCR1* and *CXCR2* mRNA expression assessed by RNA-Seq of CD34^+^ cells sorted from healthy donors (*n* = 7) and CMML patient (*n* = 12) bone marrow. Mann-Whitney *U* test. (**E**) Flow cytometry analysis of CXCR1 and CXCR2 at the surface of healthy donor (CTL, *n* = 5) and CMML patient (*n* = 18) CD34^+^ cells. Left panels, fraction of cells expressing the studied receptors; right panels, within positive cells, mean fluorescence intensity of each receptor. Mann-Whitney *U* test. (**F**) Cell output of healthy donor CD34^+^ in liquid culture in the absence or presence of 10 ng/mL CXCL8, 10 μM ladarixin (LADA), or 10 μM reparixin (REPA). Data are represented as means ± SD. *n* = 8. One-way ANOVA, Tukey’s multiple comparison. (**G**) Total colony output of healthy donor CD34^+^ cultured in methylcellulose in the absence or presence of CXCL8 (10 ng/mL), iGRAN supernatant, or reparixin (10 μM); ratio related to untreated samples. *n* = 6. Data are represented as means ± SD. One-way ANOVA, Tukey’s multiple comparison. (**H**) The same experiment was performed by using a CXCL8 neutralizing antibody (NAB, 5 μg/mL). *n* = 5. Data are represented as means ± SD. One-way ANOVA, Tukey’s multiple comparison.*P < 0.05; **P < 0.01; ***P < 0.001.

In CMML, the majority of hematopoietic stem and progenitor cells (HSPCs) are clonal, mutated cells, with a low number of residual WT cells. We evaluated the impact of the 3 main cytokines produced by iGRANs on healthy donor (cord blood and adult bone marrow) and CMML (bone marrow) CD34^+^ cells. In liquid culture, we observed a dose-dependent inhibition of cord blood CD34^+^ cell growth in the presence of CXCL8 (Figure 6A); a similar inhibitory effect was observed using adult healthy donor bone marrow CD34^+^ cells cultured with CXCL8 (Supplemental Figure 6A). In contrast, neither IL-16 nor MCP2 modified healthy donor CD34^+^ cell growth (Supplemental Figure 6B). Importantly, the 3 cytokines including CXCL8 failed to inhibit CMML CD34^+^ cell growth (Figure 6A and Supplemental Figure 6B). Together, these results indicate a specific inhibitory effect of CXCL8 on the growth of WT cells. Consistent with these results, the growth rate (36), calculated from the number of cells generated after 3 days in culture, was specifically decreased in healthy CD34^+^ cells in the presence of CXCL8. CMML cells had a similar average growth rate as compared with healthy CD34^+^ cells, although with higher intersample heterogeneity, and their growth rate remained unchanged in the presence of CXCL8 (Supplemental Figure 6C).

Methylcellulose colony formation assays also showed a decrease in colony number when healthy, cord blood, or bone marrow CD34^+^ cells were seeded in the presence of CXCL8, whereas, again, the number of colonies generated by CMML CD34^+^ cells was not affected by the presence of CXCL8 (Figure 6B). This decrease affected granulocyte-monocyte colony-forming unit (CFU-GM) and erythrocyte colony-forming unit (CFU-E) number, as observed by microscopic visualization (Figure 6C) and validated by spectral flow cytometry analysis of the colony phenotype (Supplemental Figure 6D), suggesting that erythroid and granulo-monocytic differentiation is impacted by CXCL8 treatment, with a trend for a stronger impact on erythroid lineage.

To explore further CXCL8-induced inhibition of WT CD34^+^ cell growth, we labeled these cells with cell trace to measure the number of cell divisions after 3 days in liquid cultures and calculate a proliferation index. CXCL8 did not modify the proliferation index of cord blood or healthy bone marrow CD34^+^ cells, suggesting that the difference in cell growth could be due to differences in cell death rate (Supplemental Figure 6E). In favor of this hypothesis, the decreased number of colonies in methylcellulose was associated with more annexin V^+^ cells, which became significant with serial replating (Supplemental Figure 6F).

The lack of a response of CMML CD34^+^ cells to CXCL8 compared with healthy CD34^+^ correlated with decreased expression of the CXCL8 receptors CXCR1 and CXCR2 at both the mRNA (Figure 6D) and protein (Figure 6E) levels. Using flow cytometry (Supplemental Figure 6G), we showed that the fraction of CD34^+^ cells expressing CXCR1 and CXCR2 at the cell membrane and the median fluorescence intensity of these receptors were both decreased in CMML compared with healthy donor samples.

In an attempt to counteract the negative effect of iGRAN-derived CXCL8 on WT CD34^+^ cell growth, we checked the effect of pharmacologic inhibition of CXCL8 receptors CXCR1 and CXCR2. Among the various small-molecule CXCR1/2 antagonists that are being developed clinically, 2 were tested, namely ladarixin (4-[(2R)-1-oxo-1-(methanesulfonamide)]) (37) and reparixin (*R*(–)-2-(4-isobutylphenyl)propionyl methanesulfonide) (38). Consistent with our previous results, the presence of exogenous CXCL8 in culture decreased colony-forming capacity of WT CD34^+^ cells (Supplemental Figure 6H) as well as their proliferation in liquid culture (Figure 6F). The addition of ladarixin or reparixin in combination with CXCL8 restored the proliferation and the colony-forming capacity of WT CD34^+^ cells and prevented annexin V^+^ cells in serial replating (Supplemental Figure 6I), validating the efficacy of these drugs in this setting. iGRAN supernatant added to cell cultures had the same inhibitory effect as CXCL8 alone on CD34^+^ colony-forming capacity, and blockade of CXCL8 using a CXCR1/2 inhibitor or a CXCL8 neutralizing antibody prevented the inhibitory effects of iGRAN supernatant (Figure 6, G and H). Because of CMML clonal architecture with very few WT residual CD34^+^ cells in the bone marrow (8), we could not test the ex vivo ability of CXCR1/2 inhibitors to promote the amplification of WT cells. Of note, we did not detect any effect of reparixin or CXCL8 neutralizing antibody alone on control and CMML CD34^+^ cell proliferation and differentiation (Supplemental Figure 6J). This observation is in accordance with the lack of impact of pharmacological inhibition of CXCL8 on healthy donor CD34^+^ cells (39) and the lack of hematological toxicity of reparixin in clinical trials reported so far (40, 41).

Together, CXCL8 secreted by iGRANs that accumulate in the PB of CMML patients inhibits the growth of WT CD34^+^ cells while sparing CMML CD34^+^ cells in which CXCL8 receptors are downregulated. These data suggest that, in the context of CMML, CXCL8 neutralization or CXCL8 receptor pharmacological inhibition might contribute to restoring the growth of residual WT CD34^+^ cells.

## Discussion

The overarching aim of this study was to refine the detection of immature granulocytes named iGRANs in the PB of CMML patients and to explore their pathogenic role in disease progression. We show that flow cytometry quantification of iGRANs among circulating myeloid cells provides independent prognostic information. These cells behave as myeloid-derived suppressive cells and secrete large amounts of CXCL8, an inflammatory cytokine that specifically inhibits WT CD34^+^ cells. In contrast, CXCL8 does not affect leukemic CD34^+^ cells in which CXCL8 receptors are downregulated. The ability of reparixin, an inhibitor of CXCL8 receptors, to counteract the negative effect of iGRAN supernatant on WT hematopoiesis suggests a strategy to slow down CMML evolution by reexpanding residual healthy hematopoiesis.

Flow cytometry analysis of PB cells, which supports CMML diagnosis by identifying an abnormal partition of monocyte subsets (2, 21, 22), is shown here to also be a powerful stratification tool by quantifying the iGRAN fraction or their absolute number. The cytological detection of iGRANs was suspected to define a poor prognostic subgroup of CMML patients (42), but the limited accuracy of IMC quantification on routine blood tests precluded its incorporation in the most used prognostic scores, which feature diverse combinations of age, cytopenias, and cytogenetic and molecular markers (24–26). A more precise and reproducible measurement of iGRAN fraction by flow cytometry identification of CD45^lo^, CD11b^+^, CD14^–^, CD15^+^, CD16^–^, CD24^+^, CD33^+^, CD66b^+^, and HLA-DR^–^ cells in the PB, with cutoff values at 14% of CD11b^+^CD33^+^ cells or 0.4 × 10^9^/L, may refine existing stratification scores. Besides, the accumulation of myeloid cells with a similar phenotype is a well-identified negative prognostic factor in multiple other pathological situations, including infection, trauma, and cancer (43).

The accumulation of iGRANs in the PB of a fraction of CMML patients raises questions about the mechanisms involved in their generation. The accumulation of iGRANs does not reflect subclonal genetic evolution, as these clonal cells express all the genetic variants identified in monocytes without detecting additional genomic events. The recently identified role of the chromatin regulator additional sex combs-like 1 (ASXL1) in neutrophil development, based on the neutrophilic dysplasia observed in an *Asxl1*-truncated zebrafish model (44) and the altered transcription program depicted in *Asxl1*-mutated mouse granulocyte progenitors (45), may account for the correlation between iGRAN excess and *ASXL1* gene mutation in CMML patients, both events being associated with a poor outcome (4, 19, 24–27). Some other disease features may contribute to the immunosuppressive phenotype of iGRANs. For example, the proliferative CMML subtype involves myeloid progenitor hypersensitivity to GM-CSF (9), a cytokine that promotes the generation of G-MDSCs in various other settings (43). Mutations in splicing regulator genes, which also correlate with iGRAN excess, could promote the immunosuppressive activity of G-MDSCs by activating the NF-κB signaling pathway (46, 47). Finally, iGRAN excess associates with anemia and lymphocytopenia, which may be related to the immunosuppressive potential of iGRANs (28) and their proinflammatory phenotype (48).

We show here that iGRANs are part of a dialog between clonal mature cells and HSPCs. When the occurrence of a somatic mutation in a single HSC leads to clonal outgrowth, mature myeloid cells from the clone commonly demonstrate an inflammatory phenotype and promote multiple diseases (49–51), including myeloid malignancies (52). In the context of CMML, monocytes have been shown to secrete cytokine-like 1 (CYTL1), which reduces monocyte apoptosis through an autocrine or paracrine pathway involving MCL-1 and the MAPK pathway (53), while macrophage migration inhibitory factor (MIF), which is released in the context of *TET2* truncation mutations, promotes the monocytic differentiation of HSPCs in a feed-forward loop (10). Here, we show an increased production of CXCL8 by the neutrophil lineage, correlated to the accumulation of iGRANs, which may be driven mostly by *TET2* mutation through the NF-κB pathway. Accordingly, TET2-mutated clonal hematopoiesis was associated with an increased circulating level of CXCL8 (51). The observation that the inflammatory cytokine CXCL8 specifically impacts WT CD34^+^ cell expansion and not CMML CD34^+^ is reminiscent of a zebrafish model of clonal hematopoiesis in which myeloid cells derived from mutant HSPCs secrete inflammatory cytokines that repress the growth of WT HSCs but do not affect mutated HSCs (54).

A specific decrease in the growth rate of WT CD34^+^ cells may increase the relative fitness of clonal CD34^+^ cells. The lack of the *cxcl8* gene in the mouse genome precludes the use of genetically modified mouse models to explore this hypothesis, while the clonal architecture of the disease with few residual WT cells and a growth advantage to mutated cells when undergoing differentiation (8) precludes the use of xenografted animals (47). As a heuristic tool to explore the significance of this effect, we used a mathematical model (55, 56) of the HSC compartment to estimate their fixation time (i.e., the time for one mutant to take over the compartment) either with or without CXCL8. First, we noted that, without CXCL8, the relative fitness of CMML-mutated cells (calculated from growth rates) compared with healthy CD34^+^ cells in vitro is around 1, indicating very small or even no fitness advantage and resulting in very long fixation times. Second, for the 10 out of 19 patient samples in which CMML CD34^+^ cells had a higher fitness in vitro than healthy cells (fitness > 1, Supplemental Table 7), the model predicts a drastic reduction in fixation time in the presence of CXCL8 (Supplemental Figure 6K). Last, the relative fitness of CMML CD34^+^ compared with healthy cells was always below 2 (range 1.08–1.67, Supplemental Table 7), and it is in this range that, independent of model parameterization, fixation times are most sensitive to changes in relative fitness (Supplemental Figure 6L). This suggests that, over the course of CMML development, the effect of CXCL8 on WT cells may speed up CMML clonal expansion by years to decades, which makes its inhibition an interesting clinical opportunity for patients with CMML.

Previous studies had detected high levels of multiple cytokines, including TNF-α, IL-1β, IL-6, and CXCL8, in the circulating plasma and bone marrow supernatant of CMML patients, leading to heterogeneous patterns of inflammatory protein levels (12). To date, these patterns have failed to predict the clinical response to therapeutic agents such as ruxolitinib (57). Here, 6 cytokines were found to be overproduced in the PB of patients in the iGRAN-high CMML group, of which CXCL8 was at the highest level, showed a direct correlation with iGRAN quantification, and were detected in iGRAN supernatant and by intracellular staining. These results suggest that in the heterogeneous population of CMML patients, combining iGRAN quantification by flow cytometry with circulating CXCL8 levels could define a subgroup of patients most likely to benefit from a therapeutic strategy targeting the CXCL8-mediated pathway.

In some diseases, mutant HSCs were shown to resist the chronic inflammation that otherwise triggers the exhaustion of nonmutated HSCs by switching from canonical to noncanonical NF-κB signaling (58). In the context of acute myeloid leukemia, for example, IL-1 secreted by monocytes and myeloid blast cells promotes the growth and clonogenic potential of pathogenic CD34^+^ cells while suppressing colony formation by WT CD34^+^ cells (59). Another example is the ability of *JAK2*-mutated (60) and *TET2*-mutated (61) HSPCs to resist the suppressive effect of TNF on WT HSPCs. In CMML, the absence of impact of CXCL8 on clonal cells correlates with the downregulated expression of its receptors. Given their important role in CMML pathogenesis (62), such epigenetic alterations could account for the decreased expression of CXCL8 receptors on CMML CD34^+^ cells, in contrast with other myeloid malignancies (39, 63).

Together, iGRAN excess quantified by flow cytometry appears to be an independent prognostic factor that could improve the performance of existing stratification scores in CMML. Our results indicate that CXCL8 secreted by dysplastic granulocytes specifically inhibits WT CD34^+^ cells, which may give a competitive advantage to CMML-mutated cells that have lost CXCL8 receptor expression. By relieving CXCL8 selective pressure on WT HSPCs, reparixin, an orally available inhibitor of CXCR1 and CXCR2, could modulate clonal evolution and slow down the progression of the disease. If this effect of CXCR1/2 inhibitors is validated clinically, which may be tested in the close future, its activity could be secondarily enforced by combining such an inhibitor with hypomethylating agents that reduce cell dysplasia or with cell signaling targeting drugs that decrease cell proliferation.

## Methods

### Sex as a biological variable.

Sex was not considered as a selection variable to generate cohorts of CMML patients that, in accordance with disease epidemiology (13) included more males.

### Healthy donor and patient samples.

PB samples were collected before any treatment from patients with a diagnosis of CMML according to the WHO classification. PB smears from 580 CMML patients collected from Mayo Clinic were evaluated for IMCs (IMC ≥1%), defined as myelocytes, metamyelocytes, and promyelocytes. All smears were reviewed by an expert hematopathologist. CMML samples of the learning cohort were collected between March 2015 and April 2019 from 8 French centers, and those of the validation cohort were collected independently between March 2015 and December 2021 from 10 French centers (Supplemental Table 4). Control samples were routine tube remnants (age ≥65 years, Henri Mondor Hospital Créteil, France), remnants from cytapheresis in stem cell donors (Gustave Roussy, Villejuif, France), and buffy coats from blood donors (age <65 years, Etablissement Français du Sang, Rungis, France). Bone marrow CD34^+^ cells from adult healthy donors were obtained from Lonza laboratories and the bone bank of Cochin Hospital, and umbilical cord blood samples from Saint-Louis Hospital (AC-2016-2759).

### Cell sorting.

PB samples collected on EDTA were processed within 24 hours. When indicated, blood cell stabilizer (Cytodelics, Cytodelics AB) was mixed at a 1:1 ratio to 1 mL of whole blood and transferred to a –80°C freezer. In other cases, samples were centrifuged at 300*g* for 5 minutes at room temperature (RT), plasma was collected, then PBMCs were isolated using Pancoll density centrifugation (Pan-Biotech, Dutscher). CD16^+^ neutrophils were sorted from the white cell layer directly above the RBCs using immuno-magnetic microbeads (AutoMacs System, Miltenyi Biotech). PBMCs were used for conventional flow cytometry or immuno-magnetic sorting of CD3^+^ T cells, CD14^+^ monocytes, or iGRANs (Classical Monocyte Cocktail, Miltenyi Biotec). Sorted cells (purity ≥90%) were stored at –80°C as dry pellets. iGRANs were centrifuged on microscope slides, dried for 1 hour at RT, and stained with May-Grünwald-Giemsa. Patient CD14^+^ DNA was subjected to next-generation sequencing (NGS) for a myeloid panel (3). CD34^+^ cells were sorted by AutoMacs system and frozen in FBS-DMSO 10%.

### Spectral and conventional flow cytometry.

Cryopreserved PB samples were thawed at 37°C and fixed before RBC lysis. Cells were washed and incubated with antibodies for 1 hour at 4°C, washed in BSA 1%/EDTA 0.5M in PBS, and analyzed on a CyTEK Aurora flow cytometer (Cytek Biosciences). Flow cytometry standard (FCS) files were exported using FlowJo software, version 10. Marker expression values were transformed using the auto-logicle transformation function. Phenograph clustering was performed using 28 markers and a number of nearest neighbors of 30. UMAP was run with a nearest neighbor of 15 and a minimum distance of 0.2. Conventional flow cytometry analysis was performed on 200 μL of whole blood cells using a lyse no-wash protocol (Versalyse Lysing Solution, Beckman Coulter) or on 2 × 10^6^ PBMCs labeled and analyzed using a Fortessa cytometer (BD Biosciences) and Kaluza software, version 2.1 (Beckman-Coulter). For intracellular staining, whole blood (100 μL) was diluted in 400 μL of complete medium (RPMI 1640, Gibco, Thermo Fisher Scientific) and incubated 3 hours with GolgiPlug (BD Biosciences). Cells were stained at 4°C with antibodies. After red cell lysis (1-step Fix/Lyse solution, Invitrogen), cells were permeabilized with the permeabilization buffer (Invitrogen) and stained with anti-CXCL8-PE-Cy7 (BioLegend), anti-IL-16-PE (BioLegend), or anti-MCP2-eFluor 660 (BD Biosciences). Cell death was identified by analysis of cells stained with annexin V (AnV-FITC) and propidium iodide (PI) antibodies before flow cytometry analysis (BD Biosciences).

### 3′ scRNA-Seq.

PBMCs were loaded onto a Chromium Single Cell Chip (10X Genomics), and captured mRNAs were barcoded using the Chromium Next GEM Single Cell 3′ GEM Library & Gel Bead Kit, version 3.1 (10X Genomics). Libraries were sequenced on NovaSeq 6000 (Illumina). Raw BCL files were demultiplexed using bcl2fastq (version 2.20.0.422 from Illumina) and read quality control performed using fastqc (version 0.11.9). Reads were pseudo-mapped to the Ensembl reference transcriptome v99 (homo sapiens GRCh38 build with kallisto, version 0.46.2). The index was made with kb-python (version 0.24.4) wrapper of kallisto. Barcode correction using the whitelist provided by the manufacturer and gene-based reads quantification were performed with BUStools (version 0.40.0). Empty droplets were detected using the emptyDrops function from the dropletUtils package (version 1.10.3); barcodes with *P* < 0.001 (Benjamini–Hochberg-corrected) were considered for analysis. The count matrix was filtered to exclude genes detected in less than 5 cells, cells with less than 1,500 UMIs or less than 200 detected genes, and cells with mitochondrial transcripts proportion greater than 20%. Cell-cycle scoring was performed using the CellcycleScoring function of the Seurat package (version 4.0.0) and the cyclone function of Scran (version 1.18.5). Doublets were discarded using scDblFinder (version 1.4.0) and scds (version 1.6.0). We verified that cells identified as doublets did not correspond to cells in the G2M phase. Datasets were integrated using the Harmony method, merged using Seurat (version 4.0.4), and the SCTransform normalization method was used to normalize, scale, select 3,000 highly variable genes, and regress out bias factors. The reduced PCA spaces were used as input for the HarmonyMatrix function implemented in Harmony package (version 0.1.0) where the batch effect (orig.ident) was regressed. The shared space output by Harmony was used for clustering. The optimal number of dimensions was evaluated by assessing a range of reduced Harmony spaces using 3 to 49 dimensions, with a step of 2. For space, Louvain clustering of cells was performed using a range of values for the resolution parameter from 0.1 to 1.2 with a step of 0.1. The optimal space was the combination of kept dimensions and clustering resolution resolving the best structure (clusters homogeneity and compacity) in a UMAP. Marker genes for Louvain clusters were identified through a “one versus others” differential analysis using Wilcoxon’s test through the FindAllMarkers function from Seurat, considering only genes with a minimum log fold-change of 0.5 in at least 75% of cells from one of the groups compared and FDR-adjusted *P* values < 0.05 (Benjamini–Hochberg method). UMAP visualization was done using Cerebro (version 1.2.2).

### Cytokine level measurements.

Supernatants of cultured iGRAN (24 hours) were centrifuged at 200*g* for 10 minutes and stored at –80°C. Medium without iGRAN was used as control (*n* = 3). Plasma aliquots were centrifuged at 200*g* for 15 minutes at 4°C, diluted 1:4, and analyzed using Bio-Plex Pro Human Chemokine Panel 40-Plex Assay (Bio-Rad). Acquisitions and analyses were performed on a Bio-Plex 200 system with Manager 6.1 software (Bio-Rad). Soluble S100A8/S100A9 complex (1:100) and S100A12 (1:2) were measured using R-plex Human Antibody Sets (Meso Scale Discovery), a MESO QuickPlex SQ120 reader, and the MSD’s Discovery Workbench, version 4.0. Each sample was assayed twice; average value was taken as a final result.

### Lymphocyte proliferation assay.

Ten million PBMCs were stained with anti-CD15, -CD16, -CD66b, -CD45 and -CD14 antibodies (see Supplemental Table 2, antibodies for conventional flow cytometry) before sorting CD45^+^CD15^+^CD16^–^CD66b^+^CD14^–^ cells (iGRAN) using an Influx Cell Sorter (BD Biosciences). Total PBMCs and iGRAN-depleted PBMCs were suspended in Cell Trace Violet (5 μM in 1× PBS, Thermo Fisher Scientific) for 15 minutes at 37°C, then plated in 96-well round-bottom plates (1 million/mL in complete RPMI medium). When indicated, iGRANs were added to iGRAN-depleted PBMCs (1:10 ratio). In cultures, T cells were activated in wells coated with anti-CD3 (eBiosciences, clone OKT3) and anti-CD28 (eBiosciences, clone CD28.2) antibodies in IL-2–containing medium (0.01 μg/ml, Peprotech) for 4 days. Cells were labeled with LIVE/DEAD Fixable Blue Dead Cell Stain Kit (Thermo Fisher Scientific) and antibodies and analyzed using a Fortessa.

### Cell culture and reagents.

Sorted iGRANs were cultured for 24 hours at 10^6^/mL in RPMI medium. CD34^+^ cells were cultured for 72 hours at 0.75 × 10^5^ cells/mL in complete MEM-α medium, using stem cell factor (SCF) (50 ng/mL), IL-3 (10 ng/mL), thrombopoietin (TPO) (10 ng/mL), and FMS-like tyrosine kinase 3 (FLT-3, 50 ng/mL) in the absence or presence of CXCL8 in a 37°C incubator with 5% CO_2_. All cytokines were from Peprotech. MCP2 was from Thermo Fisher, IL-16 from BioTechne. Cells were counted after Trypan blue staining. For methylcellulose assays, CD34^+^ cells were plated in duplicate at 500 cells with 1 mL complete methylcellulose (MethoCult H4034, Stem Cell) with indicated doses of CXCL8. Colonies were enumerated and phenotyped at day 14. When indicated, CXCR1/2 inhibitors reparixin and ladarixin (MedChemTronica and Clinisciences, respectively), dissolved in DMSO, Bay 11-7082 (MedChemTronica), or CXCL8 neutralizing antibody (MAB208) and mouse IgG1 isotype control (Bio-Techne) were used.

### RNA extraction, RT-qPCR analysis.

Total RNA was obtained from frozen dry pellet of sorted CD14^+^, CD3^+^ using TRIzol Reagent (Thermo Fisher Scientific) and Direct-zol RNA Miniprep (Zymo Research). For iGRAN, total RNAs were extracted using RLT buffer (QIAGEN) and TRIzol LS Reagent. Precipitated RNA was purified on a mini-RNA column (RNeasy Mini Kit from QIAGEN), quantified on Nanodrop (Spectrophotometer ND-1000), and stored at –80°C. Total RNA was reverse transcribed with SuperScript IV reverse transcriptase with random hexamers (Thermo Fisher Scientific). RT-qPCR was performed with AmpliTaq Gold polymerase in an Applied Biosystems 7500 thermocycler using standard SYBR Green detection (Thermo Fisher Scientific). Briefly, 12 ng of total cDNA, 50 nM (each) primers, and 1× SyBR Green mixture were used in a total volume of 20 μL. Primers are as follows (Thermo Fisher Scientific): *ARG1* forward: 5′-TGGGCGGAGACCACAGTT-3′; reverse: 5′-TGAGCATCCACCCAGATGAC-3′; *MPO* forward: 5′-GGAGAACGAGGGTGTGTTCAG-3′; reverse: 5′-GCCTGTGTTGTCGCAGATGA-3′; *S100A12* forward: 5′-CACATTCCTGTGCATTGAGG-3′; reverse: 5′-TGCAAGCTCCTTTGTAAGCA-3′; *TNF* forward: 5′-GGAGAAGGGTGACCGACTCA-3′; reverse: 5′-TGCCCAGACTCGGCAAAG-3′; *S100A8* forward: 5′-CAACACTGATGGTGCAGTTAACTTC-3′; reverse: 5′-CTGCCACGCCCATCTTTATC-3′; *S100A9* forward: 5′-CTGAGCTTCGAGGAGTTCATCA-3′; reverse: 5′-CGTCACCCTCGTGCATCTTC-3′; *MMP9* forward: 5′-CATCGTCATCCAGTTTGGTG-3′; reverse: 5′-AGGGACCACAACTCGTCATC-3′; *CXCL8* forward: 5′-CTGGCCGTGGCTCTCTTG-3′; reverse: 5′-CTTGGCAAAACTGCACCTTCA-3′; *RPL32* forward: 5′-TGTCCTGAATGTGGTCACCTGA-3′; reverse: 5′-CTGCAGTCTCCTTGCACACCT-3′; *GUS* forward: 5′-GAAAATATGTGGTTGGAGAGCTCATT-3′; reverse: 5′-CCGAGTGAAGATCCCCTTTTTA-3′; *GAPDH* forward: 5′-AAGGTCGGAGTCAACGGGT-3′; reverse: 5′-AGAGTTAAAAGCAGCCCTGGTG-3′; *HPRT* forward: 5′-GGACAGGACTGAACGTCTTGC-3′; reverse: 5′-CTTGAGCACACAGAGGGCTACA-3′; and P*PIA* forward: 5′-GTCGACGGCGAGCCC-3′; reverse: 5′-TCTTTGGGACCTTGTCTGCAA-3′.

### Bulk RNA-Seq.

RNA integrity (RNA integrity score ≥ 7.0) was checked on the Fragment Analyzer (Agilent), and quantity was determined using Qubit (Invitrogen). SureSelect Automated Strand Specific RNA Library Preparation Kit was used with the Bravo Platform. Briefly, 100 ng of total RNA sample was used for poly-A mRNA selection using oligo(dT) beads and subjected to thermal mRNA fragmentation before conversion into double-stranded DNA. Libraries were bar coded, purified, pooled, and paired-end sequenced on a NovaSeq 6000 sequencer (Illumina) at Gustave Roussy. Raw reads were mapped to hg19 genome with Tophat2 (version 2.0.14)/Bowtie2 (version 2.1.0). The number of reads per gene (GENECODE gene annotation v24lift37) was counted using HTSeq (0.5.4p5), and the DESeq2 (v1.10.1) package was used for differential gene expression analysis. GSEA was performed using enrichplot package with a number of permutation: 10,000, min gene set size: 20, max gene set size: 800, *P* value cutoff 0.05.

### Whole exome sequencing.

DNA collected from sorted CD14^+^, CD3^+^, and iGRANs was assayed on Nanodrop, and 200 ng genomic DNA was sheared with the Covaris E220 system (LGC Genomics/Kbioscience). Fragments were end-repaired, extended with an “A” base at the 3′ end, ligated with paired-end adapters with the Bravo platform (Agilent), and amplified for 10 cycles. Final libraries were paired-end sequenced (2 × 100 bp reads) using the Illumina NovaSeq 6000 sequencer. Somatic variants were detected in monocytes and iGRANs, using CD3 T cells as a control, and validated on IGV software, version 2.4.19.

### Statistics.

Participants’ characteristics were reported as numbers and percentages for categorical variables, mean and standard deviation (normal distribution), or median and interquartile range (skewed distribution) for continuous variables. In the absence of precision, the test is not significant. A Cox’s proportional hazards model was used to adjust the effects of iGRAN fraction (%) or absolute number (×10^9^/L) as a continuous variable on OS, defined as the time between the date of diagnosis and the date of death, whatever the cause, or censored on the date of last follow-up, and EFS, defined as the time between the date of diagnosis and date of AML transformation or death due to any cause, whichever occurs first (for patients who remain alive without AML transformation, EFS was censored on the date of last follow-up). The variables included in the GFM score (24) were used in the multivariate models (age, WBC >15 × 10^9^/L, hemoglobin <10g/L, platelets <100 × 10^9^/L, *ASXL1* mutations). The optimal cut-points were computed using maximally selected log-rank statistic (maxstat R package) (64, 65) for OS to define 2 prognostic groups, and the Kaplan-Meier method was used for survival curves (comparisons with log-rank tests). SAS 9.4 (SAS Institute Inc. Cary) and R version 4.0.5 (R Foundation for Statistical Computing) software was used.

### Study approval.

Blood samples were collected from CMML patients with informed consent within the MYELOMONO2 trial with all the needed authorizations (ethics DC-2014-2091, French Data Protection Agency, CNIL DR-2016-256).

### Mathematical modeling.

Evolution of N cells in the stem cell compartment was modeled using a Moran process (55) in which, at every iteration, one cell divides and one dies (can be the same cell), thus keeping the population size constant. The probability for a cell *i* to divide is proportional to its fitness:

(Equation 1)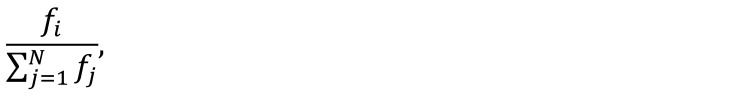 and the probability for a cell to die is 1/*N*.

To model CMML, one malignant cell with relative fitness, *r* > 1, is introduced at time *t* *=* *0* into a pool of N-1, the appropriate designation for mathematical demonstration, identical healthy cells, each with fitness 1. For an advantageous mutated cell that expands to take over the whole cell compartment, the fixation time can be approximated using the following mathematical expression:

iterations (Equation 2)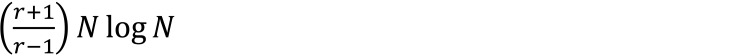 (56, 66).

We assume that the whole compartment turns over in time 1/division rate, and hence the time for one iteration is 1/(division rate*N).

The model was parameterized using in vitro experimentally derived values (for *r*) and values from the literature (for *N* and the division rate). In the basic scenario, we chose *n* = 100,000 (67) and division rate 1/year (68) (Watson, et al., ref. 67) estimate Nτ»100,000 years where 1/τ is the self-renewal/differentiation rate, with lower and upper bounds on *N* of 25,000 and 1.3 million respectively, and τ < 4years. We computed growth rates of adult healthy bone marrow (*n* = 3) and CMML (*n* = 19) CD34^+^ cells measured in liquid culture without or with CXCL8 (10 ng/mL) using an exponential model (36): 

(Equation 3)
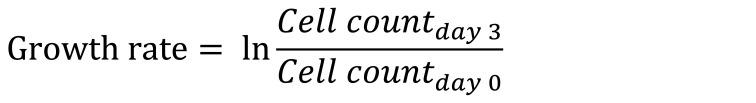


We calculated the fitness of every CMML sample as the ratio of its growth rate to the mean growth rate measured in healthy cells in the absence and presence of CXCL8. The mean growth rate of the 3 healthy samples used to compute CMML *r* was 1.17 ± 0.13 (range, 1.07-1.32) with CXCL8 and 1.54 ± 0.09 (range, 1.44-1.59) without CXCL8. As the mathematical expression for fixation time (Equation 1) requires *r* > 1, it was applied to compute the fixation time in CMML samples for which the fitness of CD34^+^ cells was higher than that measured in healthy samples (10 samples out of 19; *r* range, 1.07–1.67).

### Data availability.

scRNA-Seq, bulk RNA-Seq, and whole exome sequencing datasets generated in this study are publicly available through the European Genome-Phenome Archive (EGA) (https://ega-archive.org/) (https://ega-archive.org/datasets/EGAD50000000789). Values for all data points in graphs are reported in the Supporting Data Values file.

## Author contributions

PD, MW, AG, MM, AI, and VM acquired data. AP performed the statistical analysis. AML performed the mathematical calculations. AA and PR performed spectral flow cytometry analysis. RC, AR, and MMP analyzed data. VL, BB, OWB, CB, TB, CW, RI, PF, ST, and GE provided samples. EEM, FP, ND, and LP conducted experiments and analyzed data. LL analyzed bulk RNA-Seq experiments, conducted experiments for mathematical models, and analyzed data. DSB and ES designed research studies, conducted experiments, analyzed data, and wrote the manuscript.

## Supplementary Material

Supplemental data

Supporting data values

## Version 1 11/15/2024

Electronic publication
